# Oxygen on Arrival: An Audit of Prescription Practices in the Emergency Assessment Unit

**DOI:** 10.7759/cureus.89283

**Published:** 2025-08-03

**Authors:** Fatima Khalid, Rania M ElTahir

**Affiliations:** 1 Acute Medicine, Salford Royal National Health Service (NHS) Foundation Trust, Manchester, GBR; 2 Medicine, Allama Iqbal Medical College, Jinnah Hospital, Lahore, PAK; 3 Medicine, Salford Royal National Health Service (NHS) Foundation Trust, Manchester, GBR; 4 Medicine, Royal Colleges of Physicians, London, GBR; 5 Internal Medicine, Arab Board of Health Specializations (ABHS), Doha, QAT

**Keywords:** acute medicine, british thoracic society, clinical audit, emergency assessment unit, hospital prescribing, hypoxemia management, oxygen prescription, patient safety, prescribing compliance, target saturation

## Abstract

Introduction

Oxygen is a routinely administered, yet prescription-only therapy in hospital settings. Inappropriate prescribing can result in significant harm, particularly in patients at risk of type 2 respiratory failure. This audit aimed to evaluate adherence to guidelines on oxygen prescribing during admission clerking in the Emergency Assessment Unit (EAU) of Salford Royal NHS Foundation Trust, a UK tertiary care hospital.

Methods

A retrospective review of 100 consecutive patients admitted to the EAU over one month was conducted. Data were collected on oxygen prescription at initial clerking and throughout inpatient stay. A supplementary questionnaire assessed junior doctors’ confidence and barriers related to oxygen prescribing.

Results

Only 45% (45) of patients had oxygen prescribed at initial clerking, with a further 26% (26) prescribed later. However, 29 (29%) never received a documented oxygen prescription. Among 19 chronic obstructive pulmonary disease (COPD) patients, eight (42%) were never prescribed oxygen. Errors were found in four (9%) of prescriptions, and out-of-hours admissions were associated with higher omission rates. Of 11 junior doctor respondents, nine (82%) reported lacking formal training on oxygen prescribing, and six (55%) felt the current system did not support safe practice.

Conclusion

Oxygen prescribing during acute admissions remains suboptimal. Gaps in documentation, clinical oversight, and training-especially out of hours-contribute to non-compliance with standards. System-level changes such as mandatory electronic prompts, improved documentation processes, and targeted education are urgently needed to enhance patient safety and prescribing practices.

## Introduction

Oxygen is a commonly administered therapy in hospital settings, yet it remains a tightly regulated prescription-only medication under UK law. When used appropriately, oxygen can be life-saving by correcting hypoxemia. However, inappropriate use-particularly in patients at risk of hypercapnic respiratory failure-can result in significant harm, including carbon dioxide retention, acidosis, and death [1]. The British Thoracic Society (BTS) has issued clear guidelines for emergency oxygen use, mandating that oxygen should always be prescribed with a specified target saturation range, delivery device, and flow rate [1].

Despite such national guidelines, audits and safety reports have highlighted persistent compliance gaps. The National Patient Safety Agency (NPSA) reported that up to 27% of serious incidents involving oxygen therapy were due to prescribing or administration errors [2]. A national BTS audit found that 42.5% of patients receiving oxygen lacked a valid prescription, and 6.2% received oxygen therapy without any documentation [1].

This study evaluates oxygen prescribing practices during admission clerking for patients reviewed in the Emergency Assessment Unit (EAU) of Salford Royal NHS Foundation Trust. By measuring adherence to BTS standards, identifying omission patterns, and exploring potential causes, this audit aims to highlight areas for improvement and inform targeted interventions to enhance patient safety.

## Materials and methods

Study design

This audit employed a mixed-methods approach combining retrospective data analysis with a retrospective questionnaire to explore both compliance rates and underlying causes of non-prescription.

Data collection

Initially, electronic patient records (EPRs) were reviewed for 100 patients seen in the EAU at Salford Royal NHS Foundation Trust over a one-month period, during April 2025. Data collected included the presence of oxygen prescription on admission, the accuracy of target saturation range, and the timing of prescription (on admission vs. delayed).

A structured questionnaire was distributed to junior doctors to explore barriers to oxygen prescribing. Its content was informed by national guidelines and local audit findings, and it was pilot-tested with five junior doctors for clarity and feasibility before use. Formal validation was not performed, as the questionnaire was designed as an exploratory tool for this quality improvement (QI) audit.

We included all adult patients (aged 18 years or older) who were reviewed by the EAU clerking team during the study period. Patients were excluded if they were pediatric or were directly admitted to wards or other departments bypassing the EAU or if their medical records were incomplete or missing. As this was a retrospective clinical audit involving no patient intervention and using anonymized data, formal ethical approval was not required under NHS audit governance.

## Results

**Figure 1:**
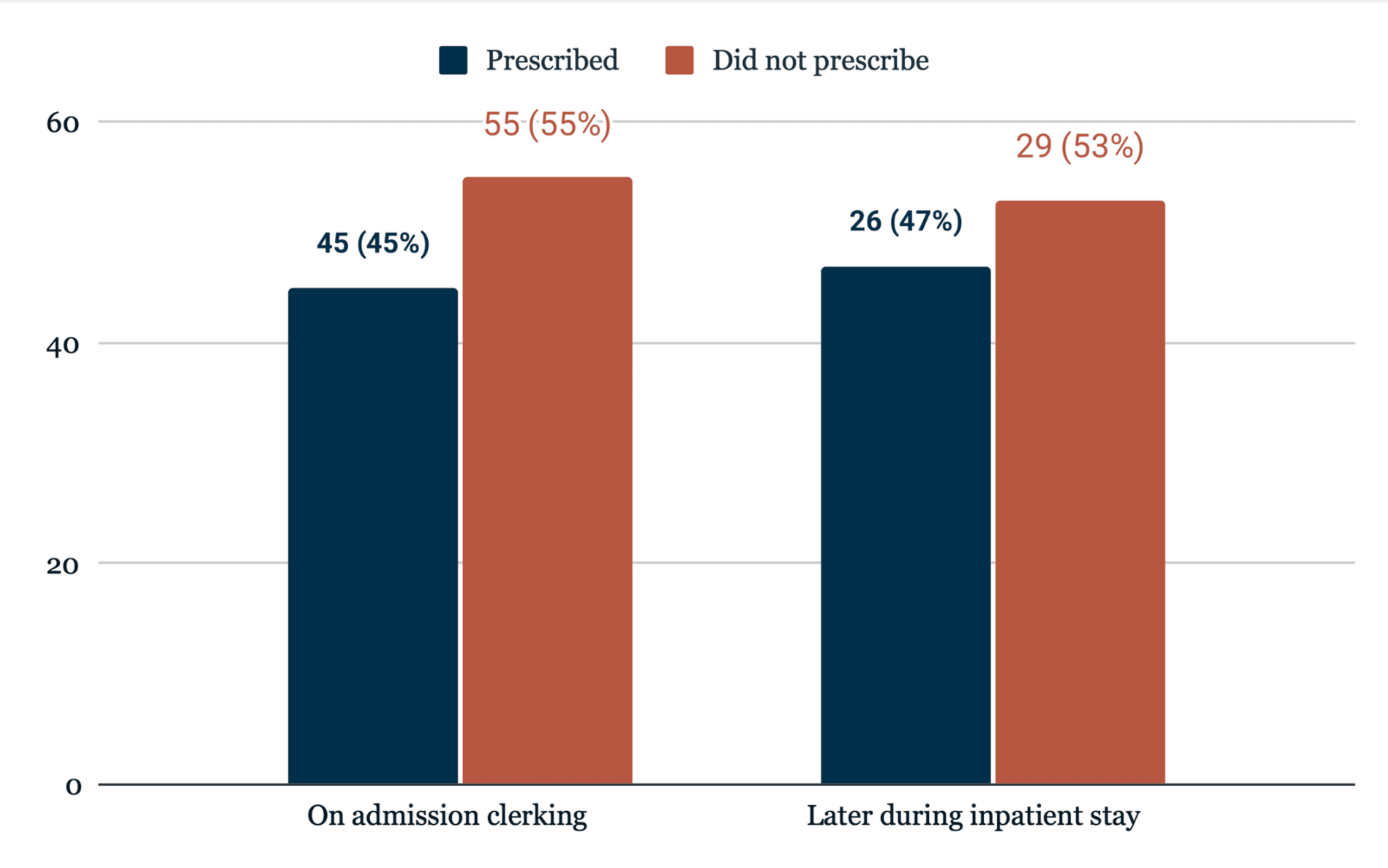
Comparison of oxygen prescription rates at initial clerking vs. during inpatient stay

A total of 100 patients admitted to the EAU during April were included in this audit. At the time of initial clerking, 45 patients (45%) were prescribed oxygen, while the remaining 55 patients (55%) had no oxygen prescription documented on admission. Of these 55 patients, 26 (47.3%) were prescribed oxygen later during their inpatient stay, whereas 29 patients (52.7%) were never prescribed oxygen at any point during admission. This comparison between prescribing at admission and later in the hospital is illustrated in Figure 1.

Among those prescribed oxygen later, 20 patients (76.9%) received their prescription within two days of admission. In the subgroup of 29 patients who were never prescribed oxygen, 24 patients (82.8%) had a hospital stay lasting fewer than five days, and five patients (17.2%) remained in the hospital for five days or longer. Notably, one of these patients had a prolonged admission of 30 days without an oxygen prescription.

**Figure 2:**
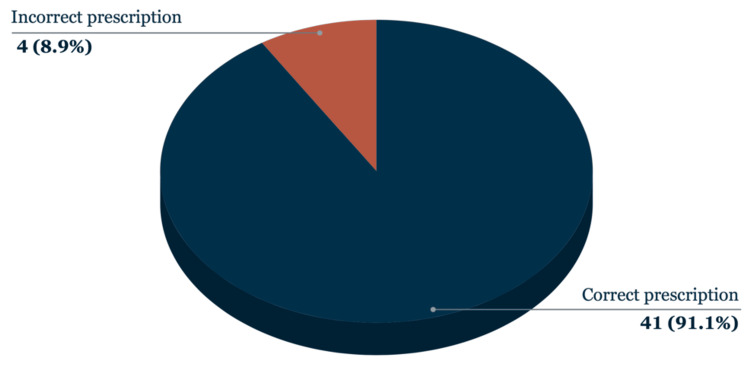
Accuracy of oxygen prescriptions

Of the 45 patients prescribed oxygen at admission, four prescriptions (8.9%) were found to be incorrect, typically due to incorrect target saturation ranges for chronic obstructive pulmonary disease (COPD) patients (Figure 2). These errors were subsequently corrected within one to four days.

**Figure 3:**
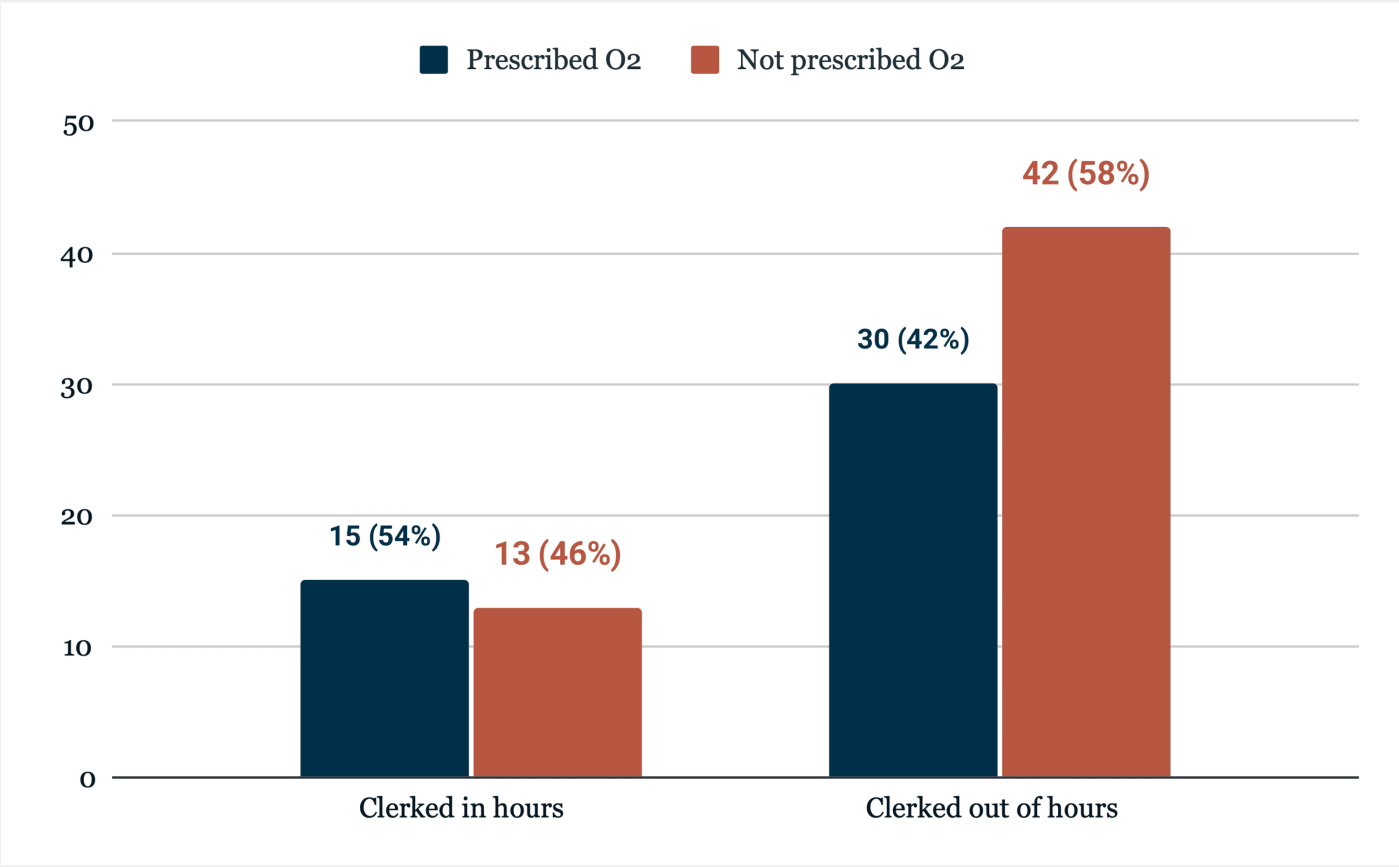
Comparison of oxygen prescription for patients clerked in hours vs. out of hours

In terms of clerking time, 72 patients (72%) were clerked out of hours. Of these, 42 patients (58.3%) did not receive an oxygen prescription on admission. In comparison, of the 28 patients clerked during standard hours, 13 (46.4%) also lacked an oxygen prescription. These patterns are depicted in Figure 3. A total of 19 patients had a documented diagnosis of COPD; notably, eight of these patients were never prescribed oxygen during their stay.

**Figure 4:**
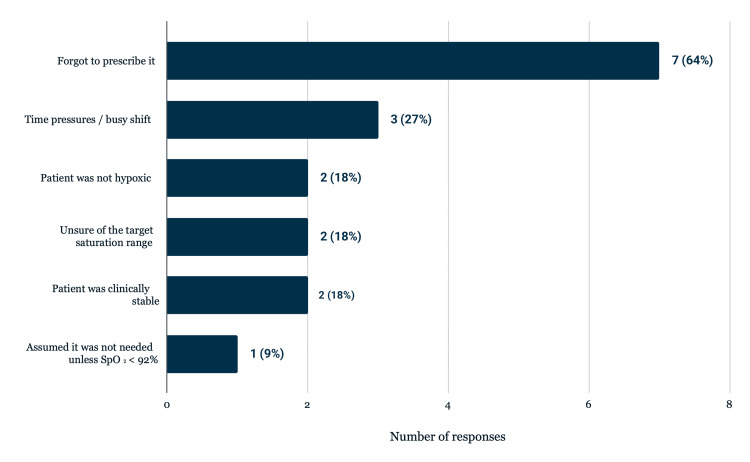
Reasons for not prescribing oxygen, total number of respondents: 11 (multiple responses allowed)

To further explore clinician behavior, 11 junior doctors completed a questionnaire on oxygen prescribing practices. Ten respondents (91%) reported that they routinely consider oxygen prescription at the time of clerking, while one (9%) did not. When asked to identify reasons for omission (multiple responses allowed), the most frequently reported barrier was “forgot to prescribe it” (n = 7, 64%), followed by time pressures or a busy shift (n = 3, 27%). Other reasons included being unsure of the target saturation range (n = 2, 18%), patients not being hypoxic (n = 2, 18%), patients being clinically stable (n = 2, 18%), and the assumption that oxygen was unnecessary unless SpO₂ was below 92% (n = 1, 9%). These findings are illustrated in Figure 4.

**Figure 5:**
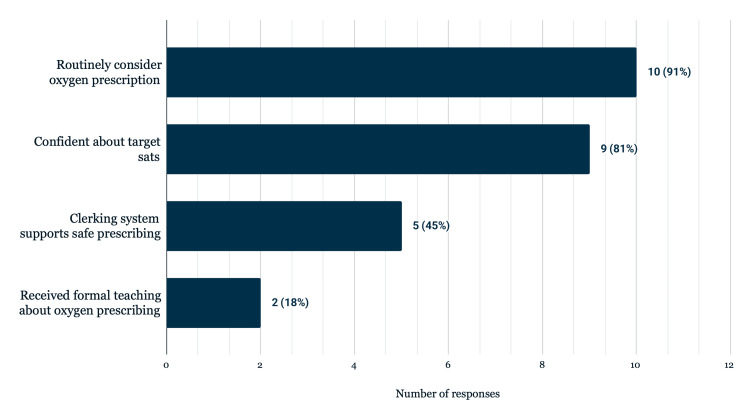
Self-reported practice and system barriers in oxygen prescribing, number of respondents: 11

Additional insights from the questionnaire revealed that nine respondents (81%) felt confident in selecting appropriate oxygen saturation targets, while two (18%) did not. Similarly, only two respondents (18%) had received formal teaching on oxygen prescribing within the past 12 months. Finally, six respondents (55%) felt that the current admission clerking proforma does not support safe oxygen prescribing, while five (45%) believed that it does (Figure 5).

## Discussion

This audit demonstrates that oxygen prescribing at the point of acute hospital admission remains inconsistent and frequently suboptimal, raising important concerns for patient safety. Our findings reveal that only 45% of patients had oxygen formally prescribed at the time of clerking. Although 26% were prescribed later, nearly one-third (29%) never had a documented oxygen order. This mirrors the national picture; the 2015 BTS Emergency Oxygen Audit reported that 45% of oxygen users lacked a valid prescription [1]. However, this figure falls short of the BTS benchmark, which mandates that 95% of patients receiving oxygen should have a documented prescription that includes a target saturation range [3].

This gap is not merely administrative. Clinically, it is significant. Oxygen, while frequently viewed as benign, is in fact a potent drug with the potential to cause harm if delivered inappropriately [4]. Both hypoxemia from under-treatment and hyperoxemia from excessive oxygen delivery have been associated with avoidable adverse outcomes. Particularly in patients with chronic lung disease, inappropriate oxygen therapy can precipitate hypercapnic respiratory failure. The NPSA has highlighted these dangers in its Rapid Response Report, identifying 281 serious incidents-including nine patient deaths-over a five-year span, largely attributed to unprescribed or improperly monitored oxygen administration [2]. These figures underscore the need to manage oxygen with the same level of care applied to any other critical drug.

While our local rate of 45% initial oxygen prescribing may appear relatively high compared to other reported rates-14% in a national audit [1], 18% in Royal Blackburn [5], and just 5% at King’s Mill Hospital [6]-it remains considerably below the ideal. Conversely, a more advanced system-wide approach at Salford Royal in a previous audit, involving automated electronic auditing, achieved an impressive compliance rate of 88.6% [7]. This contrast strongly suggests that well-designed electronic systems and QI strategies can drive better adherence to national oxygen safety standards.

Among our most concerning findings was the treatment of patients with COPD. Of the 19 COPD patients in our audit, eight (42%) received oxygen without a formal prescription. This is clinically significant, as these patients are particularly vulnerable to the harmful effects of excessive oxygen, including carbon dioxide retention, respiratory acidosis, and, in severe cases, death. National BTS guidelines clearly recommend a restricted target saturation of 88%-92% for patients at risk of hypercapnic respiratory failure to mitigate these risks [3]. Alarmingly, even among those who did receive a prescription, 9% contained errors-such as incorrect target ranges or incomplete documentation-which in some cases remained uncorrected for up to four days. This mirrors national audit data, which found that although 69% of patients had a documented target, 21.5% had saturations above it, and 8.8% of those at risk of hypercapnia were more than 2% above their upper limit [1]. These issues are not unique to our setting; Cousins et al. have similarly reported that poor oxygen prescribing and delivery practices remain widespread, often due to a lack of standardization and clinical awareness-particularly in the care of high-risk groups like COPD patients [8].

Our data also revealed a temporal trend, with oxygen more likely to be omitted during out-of-hours admissions. Of the patients clerked during evenings, nights, or weekends, 58% had no oxygen prescription documented, compared to 46% during standard working hours. This pattern likely reflects the compounding pressures of reduced staffing, time constraints, and the absence of senior supervision outside routine hours. Indeed, 64% of junior doctors surveyed acknowledged that they had forgotten to prescribe oxygen at least once, and 27% cited workload or time pressure as the reason. Although 91% of respondents reported routinely considering oxygen needs, this awareness does not consistently translate into action-indicating that human error in high-pressure environments remains a critical barrier to safe practice.

A major contributing factor is likely the lack of formal education around oxygen prescribing. In our survey, 82% of junior doctors reported receiving little or no training on the topic. This finding aligns with previous literature: Ganeshan et al. identified substantial knowledge gaps among junior doctors, many of whom failed to select appropriate oxygen delivery methods or target ranges in simulated scenarios [9]. This is further compounded by structural issues in documentation. Over half of our junior doctor respondents felt that the current system did not adequately support safe oxygen prescribing-often due to non-mandatory or poorly integrated fields for oxygen on clerking proformas or electronic drug charts. While BTS guidance urges hospitals to record target saturations for all admitted patients, regardless of current oxygen need [3], real-world compliance has been inconsistent. A 2015 audit showed that even though 70% of hospitals had a formal oxygen prescribing policy, implementation was frequently lacking [1].

Importantly, there is clear evidence that targeted QI interventions can markedly enhance compliance with oxygen prescribing standards. A multifaceted initiative described by Ranjbar and Dorai at a UK teaching hospital involved redesigning drug charts to include mandatory oxygen sections, adopting the NEWS2 early warning score (which flags hypoxemia), providing mandatory training for junior doctors, displaying educational posters, and issuing regular handover prompts [10]. These measures led to substantial improvements: unprescribed oxygen use fell to 14%, and nearly all patients on oxygen had a documented target range [10]. Similarly, Hussain et al. reported that introducing visual reminders and structured handover tools increased compliance from 18% to 54.8%, and among patients requiring oxygen, prescription rates improved to 90.9% [5]. Additional audit data indicate that targeted reminders and pre-filled electronic prescription templates further improve valid prescription rates [11,12]. Complementing these findings, a human factors study at a UK teaching hospital applied the Functional Resonance Analysis Method (FRAM) to map real-world prescribing workflows, identify hidden system barriers, and develop sustainable interventions-such as redesigned electronic forms and context-aware prompts-that significantly enhanced oxygen prescription reliability [13].

Electronic prescribing systems are powerful but require embedded reminders. Without prompts, they do not necessarily improve prescribing. We recommend implementing electronic “forcing functions” within our e-prescribing system. These could include mandatory oxygen target fields in admission clerking templates, automatic alerts when high-risk patients (such as those with COPD) are admitted without a documented target, and mandatory fields requiring justification for omitting oxygen prescriptions. Such changes should be paired with structured induction teaching for new doctors, focused educational interventions for all prescribing staff, and clear, visible prompts integrated into the clinical workflow. Additionally, continuous audit cycles-with feedback to teams-can sustain awareness and drive further improvements.

While this audit provides valuable insights into oxygen prescribing practices, several limitations must be acknowledged. First, as a single-center study, our findings may not be fully generalizable to other hospitals with different workflows, staffing levels, or electronic prescribing systems. Second, we did not assess clinical outcomes directly, such as rates of hypoxemia or hypercapnic respiratory failure, so the impact of prescribing omissions on patient morbidity or mortality remains inferred rather than measured. Third, our survey of junior doctors, while informative, was subject to response and recall bias and may not capture the full range of clinician perspectives. Future audits could benefit from a prospective design, inclusion of patient outcomes, and multicenter data to enhance robustness and external validity.

## Conclusions

This audit highlights that oxygen prescribing during acute admissions remains inconsistent and below recommended standards, with potential implications for patient safety. Contributing factors include human error under time pressure, lack of formal training, and suboptimal integration of oxygen prescribing into clinical workflows. To address these gaps, a multifaceted approach is required, combining staff education, system-level changes such as electronic prompts and mandatory documentation fields, and regular audit with feedback. Implementing these strategies will help ensure that oxygen therapy is prescribed safely and appropriately for all patients.

## Figures and Tables

**Figure 6:**
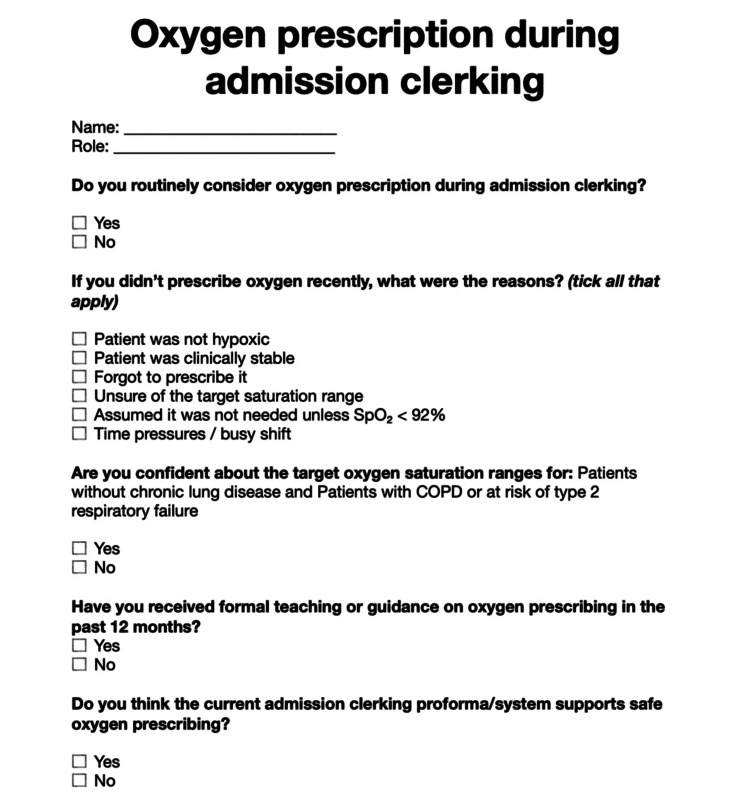
Survey questionnaire
